# Amyloid-Beta Influences Memory *via* Functional Connectivity During Memory Retrieval in Alzheimer's Disease

**DOI:** 10.3389/fnagi.2021.721171

**Published:** 2021-09-01

**Authors:** Binyin Li, Miao Zhang, Ikbeom Jang, Guanyu Ye, Liche Zhou, Guiying He, Xiaozhu Lin, Hongping Meng, Xinyun Huang, Wangxi Hai, Shengdi Chen, Biao Li, Jun Liu

**Affiliations:** ^1^Department of Neurology and Institute of Neurology, Ruijin Hospital, Shanghai Jiao Tong University School of Medicine, Shanghai, China; ^2^Department of Nuclear Medicine, Ruijin Hospital, Shanghai Jiao Tong University School of Medicine, Shanghai, China; ^3^MGH/MIT/HMS Athinoula A. Martinos Center for Biomedical Imaging, Massachusetts General Hospital, Boston, MA, United States; ^4^Department of Radiology, Harvard Medical School, Boston, MA, United States; ^5^Collaborative Innovation Center for Molecular Imaging of Precision Medicine, Ruijin Center, Shanghai, China

**Keywords:** amyloid, magnetic resonance imaging, Alzheimer disease, memory, neural connectivity

## Abstract

**Objective:** Amnesia in Alzheimer's disease (AD) appears early and could be caused by encoding deficiency, consolidation dysfunction, and/or impairment in the retrieval of stored memory information. The relationship between AD pathology biomarker β-amyloid and memory dysfunction is unclear.

**Method:** The memory task functional MRI and amyloid PET were simultaneously performed to investigate the relationship between memory performance, memory phase-related functional connectivity, and cortical β-amyloid deposition. We clustered functional networks during memory maintenance and compared network connectivity between groups in each memory phase. Mediation analysis was performed to investigate the mediator between β-amyloid and related cognitive performance.

**Results:** Alzheimer's disease was primarily characterized by decreased functional connectivity in a data-driven network composed of an *a priori* default mode network, limbic network, and frontoparietal network during the memory maintenance (0.205 vs. 0.236, *p* = 0.04) and retrieval phase (0.159 vs. 0.183, *p* = 0.017). Within the network, AD had more regions with reduced connectivity during the retrieval than the maintenance and encoding phases (chi-square *p* = 0.01 and < 0.001). Furthermore, the global cortical β-amyloid negatively correlated with network connectivity during the memory retrieval phase (*R* = – 0.247, *p* = 0.032), with this relationship mediating the effect of cortical β-amyloid on memory performance (average causal mediation effect = – 0.05, *p* = 0.035).

**Conclusion:** We demonstrated that AD had decreased connectivity in specific networks during the memory retrieval phase. Impaired functional connectivity during memory retrieval mediated the adverse effect of β-amyloid on memory. These findings help to elucidate the involvement of cortical β-amyloid (Aβ) in the memory performance in the early stages of AD.

## Introduction

Alzheimer's disease is marked by prominent memory deficiency in the early stage. It is controversial whether the observed amnesia is due to disrupted encoding, the consolidation of episodic information, or an impairment in the retrieval of stored memory information. Several studies have suggested that the ineffective encoding of new information in the brain triggers episodic memory deficits (Pasquier et al., 2001; Weintraub et al., 2012), while more evidence supported a retrieval deficit in early Alzheimer's disease (AD) (Grober and Kawas, 1997; Albert et al., 2001; Li et al., 2016). Transgenic AD mouse models also suggested that the direct activation of hippocampal memory engram cells resulted in memory retrieval, revealing a major impairment of retrieval, rather than storage, in early AD (Roy et al., 2016).

Many studies have been conducted to show that memory is retrieved by neural networks (Palm, 2013). The integration between neural networks supports autobiographic memory retrieval, with network connectivity increasing when memories are accessible and recollected (St Jacques et al., 2011). In AD, the inability to encode and retrieve memory is present at the earliest stage of the disease and deteriorates throughout the course of the disease (Aggarwal et al., 2005). The aggregated β-amyloid (Aβ), which accumulates as the first pathological marker of AD, was involved in neural network dysfunction (Palmqvist et al., 2017). Clinically, episodic memory processing-related medial temporal lobe activation was negatively associated with higher global Aβ levels (Mormino et al., 2012; Vannini et al., 2012).

Together, these findings suggest that Aβ pathology might exert an influence on neural network dysfunction, and this disruption causes worse memory performance in AD. However, this hypothesis has not been tested in humans. To address this caveat in the literature, detailed alterations in cortical connectivity networks during different memory phases (encoding, maintenance, and retrieval) in 36 patients with AD and 36 normal participants by task functional MRI (fMRI) were characterized. Simultaneously, cortical Aβ deposition were measured using PET. The level of Aβ pathology in the cortex was evaluated for its effect on memory-related cortical functional networks. To assess the clinical relevance of the observed functional alterations, the Aβ pathologies were related to measures of memory performance.

Our primary hypothesis was that AD functional connectivity changed within specific cortical networks during memory retrieval. With this, the cortical Aβ deposition would correlate with the functional connectivity, and this relationship would mediate the influence of cortical Aβ on memory performance. The findings would further our understanding of the mechanisms underlying memory deficiency in AD and the effect of Aβ pathology on memory-related functional neural networks.

## Materials and Methods

### Participants

In the cohort recruitment, AD was diagnosed using the syndromal categorical cognitive staging scheme and positive amyloid deposition by PET, based on the National Institute on Aging-Alzheimer's Association workgroups (Jack et al., 2018). According to cognitive status, neuro psychologic test performance, and Aβ positivity determined by an agreement between one nuclear medicine specialist and one memory-disorder specialist, 36 Aβ positive (Aβ+) AD participants and 36 Aβ negative (Aβ-) normal controls were included in this study.

All participants underwent neuropsychological tests that included the Mini-Mental State Examination (MMSE, Chinese Version) (Katzman et al., 1988), global clinical dementia rating (CDR > 0.5 for AD diagnosis), Zung Self-rating Anxiety Scale, Self-rating Depression scale, Activities of Daily Living questionnaire (for caregivers), the Beijing version of the Montreal Cognitive Assessment (MoCA), and the Chinese version of Addenbrooke's Cognitive Examination-Revised (ACER) with subtests of memory, language, attention, fluency, and visual-spatial processing (Fang et al., 2014).

The present study was approved by the Ethics Committee (internal review board) of the Shanghai Jiao Tong University-affiliated Ruijin Hospital, China. According to the Declaration of Helsinki, all participants in the study or their caregivers signed written informed consent before being included.

### Task Design in fMRI

**Figure 1:**
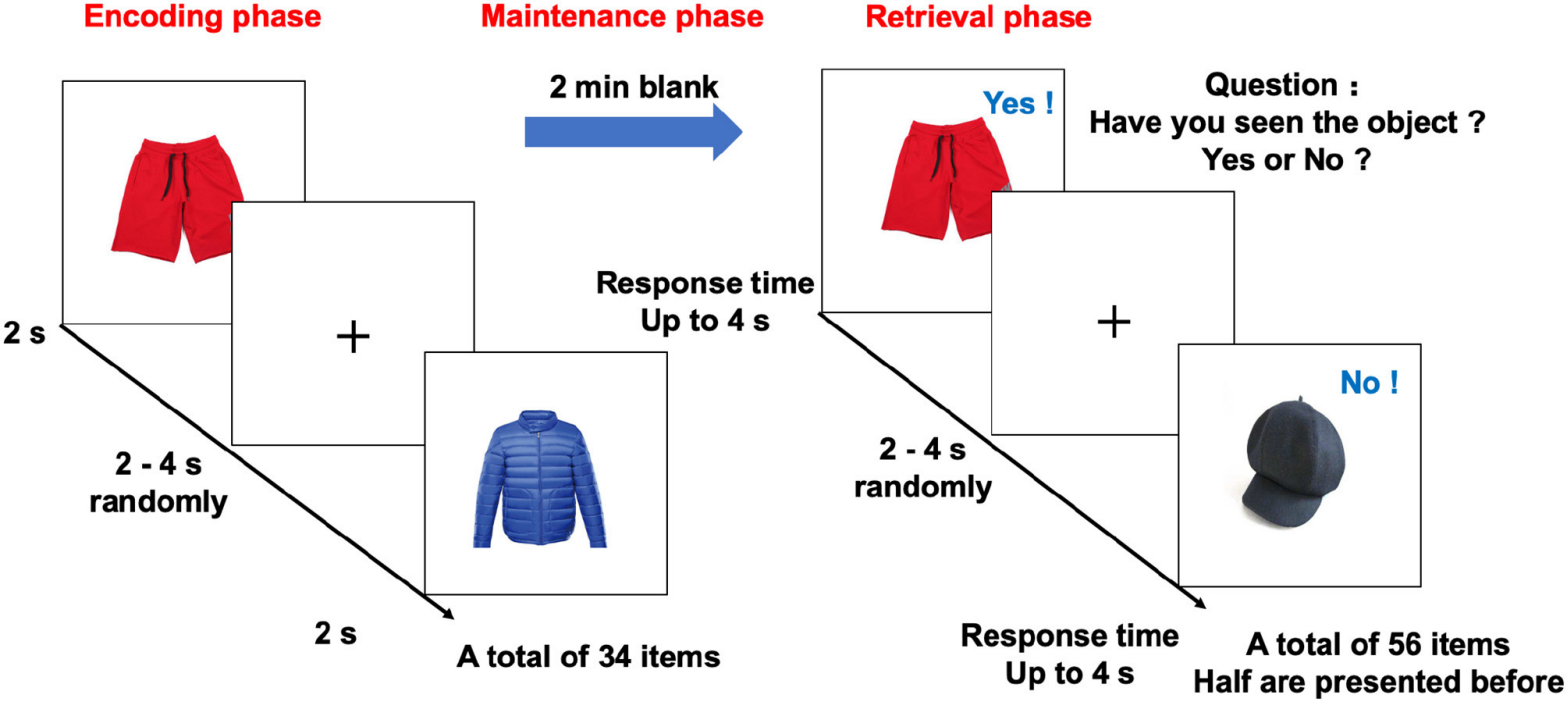
Design of the memory task in the fMRI study. The memory task in the fMRI study, included encoding, blank (memory maintenance), and retrieval phases. In the encoding phase, 34 objects were sequentially presented for 2 s. After a 2-min blank, the participants indicated whether the object was present or absent in the encoding phase as quickly as possible using two buttons of an MR-compatible button box. During retrieval, each object appeared on the screen until there was a response or up to 4 s before the next letter was shown. The inter-object interval was randomly set between 2 and 4 s. fMRI, functional magnetic resonance imaging.

The task used an event-related design, with a total of 34 encoding objects and 56 retrieval objects (Figure 1). During the encoding phase, the participants studied 34 unique objects (with a 3 ×3 degree visual angle), which were presented in pairs and had 2–6 randomized interstimulus intervals. In the following 2-min maintenance phase, the screen was blank and no stimulus was presented. Then, in the retrieval phase, there were 56 test trials that presented objects with the same visual angle. The objects were presented sequentially, followed by 2- to 6-s interstimulus intervals. The participants were asked to judge whether the objects were “old” (studied) or “new” (unstudied). The participants had up to 4 s to respond. Among these trials, half presented old (studied) objects and half presented new (unstudied) objects. Task accuracy was defined as the percentage of the correct answer in 56 recognition objects.

The participants viewed an MRI-compatible display monitor *via* a mirror mounted to the head coil. All responses were recorded by an MRI-compatible optical mouse. The order of object presentation was randomized across all participants. The duration of the task slightly varied from 11 to 13 min because of the interstimulus interval randomization and different reaction times.

### Acquisition of MR and PET Images

The MRI data were acquired on a whole-body PET–MR scanner (Biograph mMR; Siemens Healthcare, Erlangen, Germany). Blood oxygen level-dependent (BOLD) MRIs using echo-planar imaging weighted sequence were performed during the tasks (260 functional images, 3,000-ms repetition time, 30-ms echo time, matrix = 64 ×64, a 90° flip angle, 35 slices, no slice gap, and a voxel size of 3 ×3 ×3.75 mm^3^). The T1-weighted three-dimensional structural image was acquired with TR = 1,900 ms, TE = 2.44 ms, and 192 slices covering the whole brain.

The PET scans were performed simultaneously using an ^18^F florbetapir (AV45) tracer to image Aβ. The participants received an intravenous injection of AV45 at a mean dose of 3.7 MBq/kg body weight after finishing the task fMRI. Then, static AV45-PET data were acquired in sinogram mode 50 min after injection using the following parameters: 128 slices (gap, 0.5 mm) covering the whole brain, a matrix size of 344 ×344, a voxel size of 2.6 ×2.6 ×3.1 mm^3^, reconstructed with high-definition PET (21 subsets, 4 iterations), and post-filtered with an isotropic full-width half-maximum (FWHM) Gaussian kernel of 2 mm. The attenuation correction for PET was performed using MR-based attenuation maps derived from a dual-echo Dixon-based sequence.

An automatic pipeline was employed to extract cortical standardized uptake value ratios (SUVRs) by PETSurfer FreeSurfer 6.0 (https://surfer.nmr.mgh.harvard.edu/fswiki/PetSurfer) using the cerebellum cortex as the reference region. In detail, structural T1 images were used to create a high-resolution segmentation using the Schaefer function atlas (Schaefer et al., 2018) to run the partial volume correction (PVC) methods. The PET/anatomical image registration was then performed and visually checked. To minimize the partial volume effect from cortical atrophy in AD, the extended Muller–Gartner method as a PVC method (Müller-Gärtner et al., 1992; Greve et al., 2016) was applied. Surface-based SUVR maps were smoothed on the two-dimensional surface by a Gaussian kernel of 5 mm in FWHM.

### fMRI Activation Analysis: Standard Univariate Analysis

Functional and T1 MRI data were pre-processed by Freesurfer v6.0 and FsFast v5.0 (surfer.nmr.mgh.harvard.edu). The pre-processing pipeline for fMRI included templating from the middle time point of raw functional data, masking, functional-anatomical registration, motion correction, slice-timing, and resampling the raw time courses to the left and right hemispheres. Quality control of functional-anatomical registration was performed by both the automatic rating from Freesurfer and visual inspection. Two-dimensional spatial smoothing was performed for surface data with a Gaussian kernel of 5 mm in FWHM.

The encoding and retrieval phases were evaluated separately using a surface-based stream. For the first-level general linear model (GLM) analysis (within-subject), contrasts (encoding stimulus against interstimulus rest and recognition stimulus against interstimulus rest) were calculated by the gamma hemodynamic response function within the cortical surface at each voxel. Each case was inspected after the first-level analysis by visualization to ensure that they registered well with the FreeSurfer average surface (common space). All cases were concatenated for further analysis.

In the second level (between-subject) analysis, task accuracy-related activation maps were generated in all participants by GLM with task accuracy as a regressor. Significant clusters were computed after permutation resampling by bootstrapped Monte Carlo simulations (10,000 iterations) at *p* = 0.001 to correct for multiple comparisons across all brain voxels. Finally, we collected clusters of that size or larger during the simulation and corrected them to a threshold of *p* < 0.05 (Greve and Fischl, 2018). The coefficients of activation were extracted after mapping on the native surface using an inverse affine transform for further analysis.

### Memory Phase-Related Time Courses

We re-preprocessed the fMRI by the pipeline implemented in the CONN toolbox (version19.c) (https://www.nitrc.org/projects/conn) within MATLAB, similar to a previous study (Nour et al., 2019). This pre-processing included slice timing correction, the realignment of functional scans and normalization to MNI space, and spatial smoothing (Gaussian kernel of 8 mm in FWHM). In the denoising step, linear regression was used to remove the influence from (1) the BOLD signal from the white matter and CSF voxels (five components each, derived using the anatomical component-based correction implemented using the ART toolbox), (2) six residual head motion parameters and their first order temporal derivatives, (3) the scrubbing of artifact or outlier scans, and (4) the effect of task-condition using event regressors (encoding and retrieval stimulus) convolved with the hemodynamic response function. Finally, the de-noising step included temporal bandpass filtering (0.008–0.09 Hz) and the linear de-trending of the time courses.

Following pre-processing, the whole BOLD time courses were split into three memory phases (encoding, maintenance, and retrieval) for each participant and then extracted individual mean time courses within 200 cortical nodes, which were defined by the Schaefer function atlas (Schaefer et al., 2018). The 200 nodes can be assigned to seven *a priori* functional networks, namely, the visual network, somatomotor network, dorsal attention network (DAN), ventral attention network (VAN), limbic network, frontoparietal network (FPN), and default mode network (DMN).

### Data-Driven Detection of Empirical Networks

The functional connectivity between two brain regions (nodes) was defined as the Fisher z-transformed Pearson's correlation coefficient between the mean BOLD time course in each region (Rubinov and Sporns, 2010). The functional connectivity in the maintenance phase from the control group was used to generate an undirected weighted covariance matrix, *C*, where *c*_*ij*_ represents the functional connectivity between nodes *i* and *j* (i.e., edge strength between nodes). After setting negative weights to 0, the Louvain algorithm (implemented in the MATLAB Brain Connectivity Toolbox, https://sites.google.com/site/bctnet/) was used to get the control group-level empirical networks (Bostanciklioglu, 2020) in the maintenance phase (see Supplementary Material).

The community detection analysis was restricted to the 136 nodes allocated to the limbic network, DMN, DAN, VAN, and FPN suggested by a previous cognition study (Nour et al., 2019). Given the stochastic nature of the Louvain algorithm, a consensus clustering approach was used to ensure the robustness of the final community structure by the Agreement and Consensus functions in the Brain Connectivity Toolbox (Blondel et al., 2008, Cohen and D'Esposito, 2016, Hearne et al., 2017) (see Supplementary Material for details). Finally, these nodes were clustered into three new empirical networks from controls.

### Empirical Network Connectivity Comparison in Each Memory Phase

The network component analysis was performed using a node-wise connectivity comparison between the networks, which showed group differences in the mean functional connectivity. For functional connectivity in each phase, it entered into a group analysis that gauged the differences in AD and controls using codes similar to the NBS (v1.2) algorithm (Zalesky et al., 2010). Different from the traditional NBS, all the *t*-values of the links were calculated in a general linear model after regressing out age and sex (Berron et al., 2020). Applying a two-sided suprathreshold, the differences in node–node functional connectivity between two groups were visualized as binary outcomes in each memory phase in the BrainNet viewer (Xia et al., 2013).

The BOLD information content was also gauged using an entropy measure for discrete-time courses called sample entropy (Richman and Moorman, 2000) (setting embedding dimension m = 2 and the range in standard deviation from all-time courses). The measure should be higher for time courses with lower predictability and random disorder and conversely reduced for more ordered and predictable time courses (Yao et al., 2013).

### Statistical Analyses

Statistical analyses were performed by R (Version 3.6.2). The AD and controls were compared using an independent *t*-test or chi-square test for continuous or nominal variables, respectively. Pearson's correlation was used to test the relationships between connectivity in each phase and cortical Aβ SUVR. All significant results were double-checked by the *RVAideMemoire* package in R, performing a resampling test with 10,000 permutations.

To explore the effect of Aβ on cognition, General Linear Regressions was further used to set network connectivity and cortical amyloid as predictors of cognitive performance, adjusted for age, education, and sex. The hypothesis that cortical Aβ influenced memory performance was tested *via* modulating network connectivity by a mediation analysis using the “mediation” package in R, implementing a non-parametric bootstrap method with bias-corrected and accelerated confidence intervals and 10,000 simulation draws.

All tests were two-tailed, and values of corrected *p* < 0.05 were considered statistically significant unless specified otherwise. Supplementary Figure 1 summarizes our analysis approach.

## Results

### Participants and Task-Related Activation

A total of 36 AD and 36 normal controls satisfied the criteria and were included. The two groups were matched in age, education, and sex (Table 1), and AD had worse cognitive performance in all cognitive domains assessed by the MMSE, MoCA, and ACER, as expected. In the fMRI memory task, the AD group had lower accuracy than the control group. Furthermore, task accuracy had significant correlations with the MoCA (*R* = 0.61, *p* < 0.001), ACER (*R* = 0.652, *p* < 0.001), and ACER memory scores (*R* = 0.696, *p* < 0.001).

**Table 1:** Demographic and clinical characteristics.

	**AD**	**Control**	**P**
Number	36	36	
Sex (M/F)	17/19	15/21	0.232
Age	68.78 ± 7.63	69.05 ± 7.11	0.778
Education	12.08 ± 2.53	13.27 ± 2.83	0.076
MMSE	19.88 ± 5.49	28.05 ± 1.86	<0.001
MoCA	15.36 ± 6.71	26.54 ± 2.41	<0.001
ACER	56.50 ± 18.8	89.30 ± 6.73	<0.001
Attention	11.61 ± 3.81	17.02 ± 1.13	<0.001
Memory	15.42 ± 4.92	21.61 ± 3.73	<0.001
Fluency	6.19 ± 3.38	10.11 ± 2.15	<0.001
Language	19.00 ± 5.74	24.97 ± 1.68	<0.001
Visuospatial	11.13 ± 4.69	15.72 ± 0.55	<0.001
Task accuracy	0.23 ± 0.09	0.62 ± 0.38	<0.001
Cortical thickness	2.28 ± 0.15	2.39 ± 0.11	<0.001

*Data are expressed as the mean ± standard deviation. A two-sample independent permutation t-test was conducted to test between-group differences. The chi-square test was used for categorical variables. ACER, Addenbrooke's Cognitive Examination-Revised; MoCA, Montreal Cognitive Assessment*.

**Figure 2:**
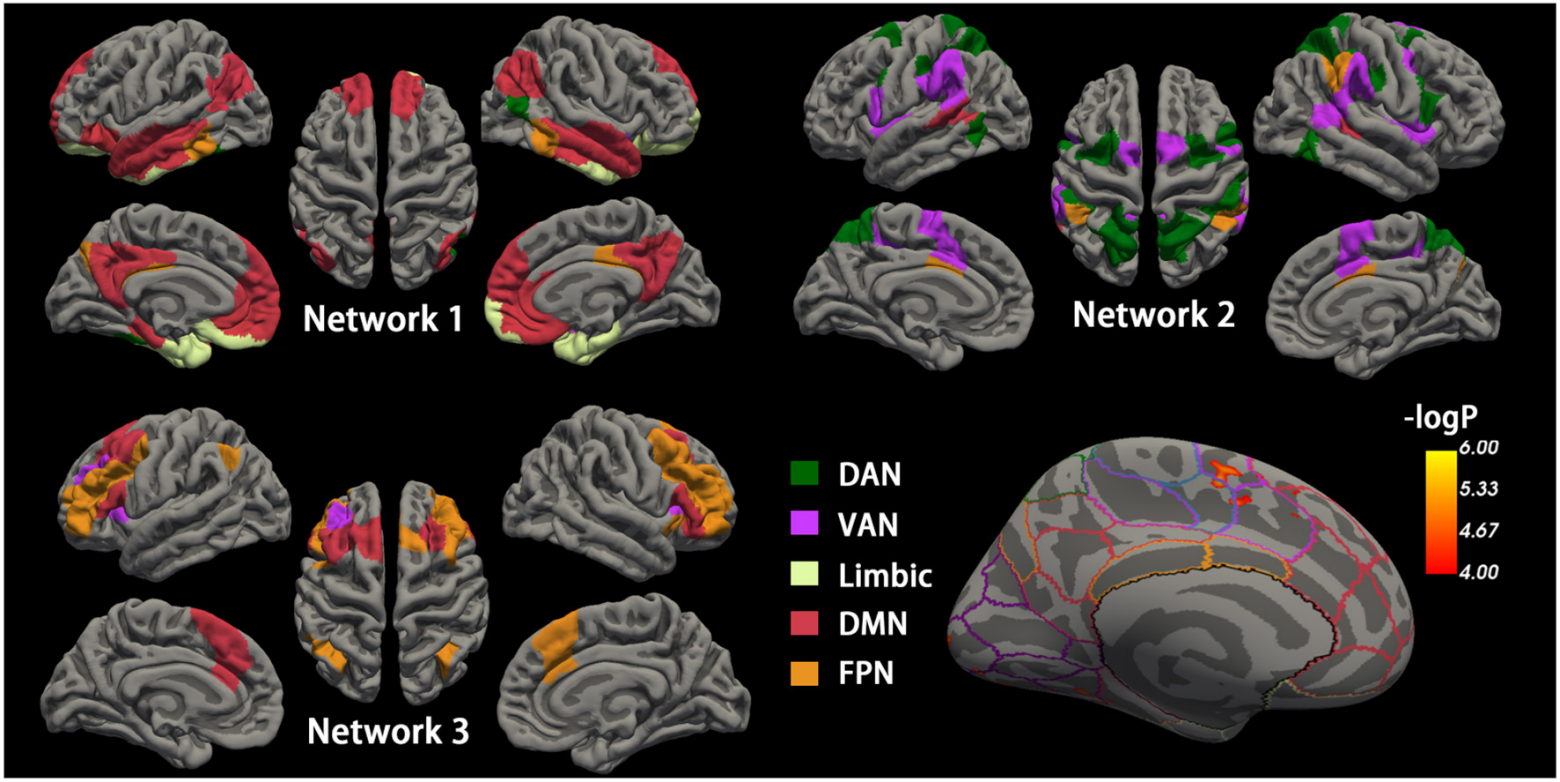
Cortical networks during the maintenance phase and accuracy-related neural activation. Top-left, top-right, and bottom-left cortical mappings illustrated the nodes of the three empirical networks identified by the community detection algorithm during the maintenance phase, colored by *a priori* network assignments from the Schaefer cortical parcellation. Network 1 is mainly composed of the DMN and limbic network, and Network 2 consists of DMN and FPN. Network 3 generally includes DAN and VAN. The bottom right shows the surface maps of the activated cortex in the retrieval phases related to accuracy. The border on the surface indicates the network identical to the above Schaefer cortical parcellation. The activation is observed in the region of VAN. DMN, default mode network; FPN, frontoparietal network; DAN, dorsal attention network; VAN, ventral attention network.

As expected, in the standard fMRI GLM analysis, there were widespread activations associated with task accuracy in the visual, frontoparietal, and attention networks of the left hemisphere during memory retrieval (voxel-wise *p* < 0.001 and cluster-wise corrected *p* < 0.05 after correction by randomized permutation simulator). Besides the visual network, the most prominent activation was in the left superior frontal cortex (Talairach Montreal Neurological Institute coordinates: −5.8, −7.8, 53.5; *p* = 0.001, activation β = 5.429, lower right in Figure 2). It was located in the ventral attention network, and mainly correlated with ACER memory (*R* = 0.435, *p* < 0.001), ACER (*R* = 0.348, *p* < 0.001), and MoCA (*R* = 0.373, *p* < 0.001). In contrast, in the encoding phase, there was no significant linear or quadratic relationship between neural activation and task accuracy in the second-level voxel-wise analysis.

### Empirical Networks From *a priori* Network Assignments

To investigate memory phase-related functional connectivity changes within task-relevant cortical networks, a data-driven community detection algorithm (Blondel et al., 2008, Lancichinetti and Fortunato, 2012) was first used to partition the 200 cortical nodes into three group-level networks: Network 1 consisting of 56 nodes, Network 2 with 50 nodes, and Network 3 with 30 nodes (Figure 2). All three empirical networks were visually symmetrical in the cortex. The “empirical” Network 1 node assignments overlapped with the *a priori* network assignments from the Schaefer functional atlas in DMN, limbic network, and FPN. Network 2 mainly overlapped with the dorsal and ventral attention networks and the spatial activation maps from the standard fMRI activation analysis. Network 3 generally included regions assigned to FPN and its adjacent DMN regions.

**Figure 3:**
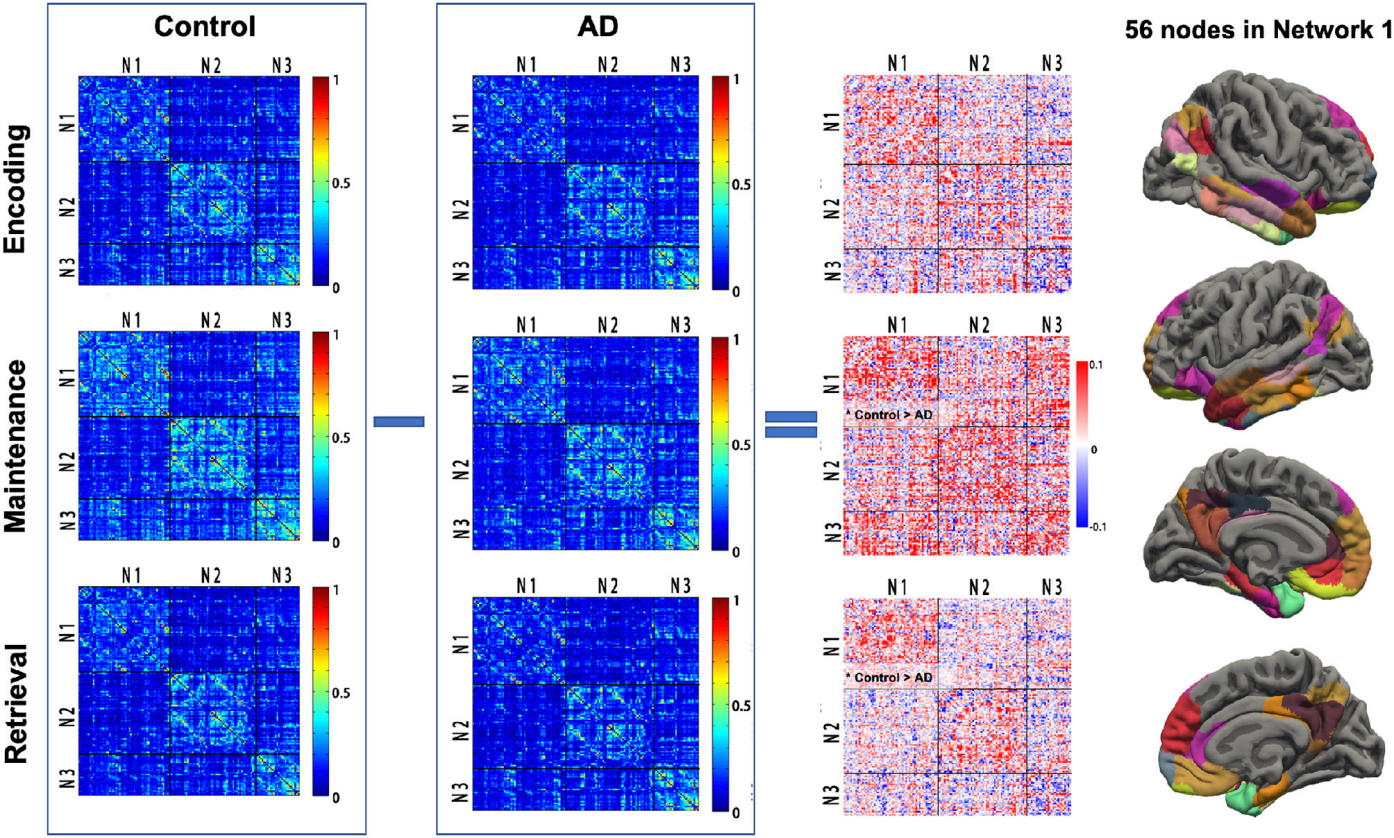
Task phase-related functional connectivity in AD and controls. The first and second columns: averaged functional connectivity (FC) matrices in each task phase of controls and AD. Each cell of the matrix represents the FC strength (Fisher *z*-transformed *r*-value) between the cortical nodes. The third column: The difference of FC (control—AD) in each task phase between AD and controls. Black lines indicate the boundary separating nodes allocated to the empirical Networks 1–3. The fourth column illustrated the 56 cortex nodes in Network 1. *Control > AD, significantly reduced mean FC in the Network 1-Network 1 matrix in AD; AD, Alzheimer's disease; N1, Network 1; N2, Network 2; N3, Network 3.

For each participant, the phase-related functional connectivity was defined as the connectivity between two nodes during the phase. Thus, each participant had three connectivity matrices within each network and three matrices between networks (i.e., Network 1–Network 2, Network 2–Network 3, Network 1–Network 3). The mean matrices for the AD and control group are shown in Figure 3.

### Connectivity Within Network 1 Is Reduced in AD

The group difference of the mean functional connectivity within the three empirical networks between the AD and controls was first investigated. The AD group had significantly reduced connectivity within Network 1 during maintenance (0.205 vs. 0.236, *p* = 0.04) and retrieval (0.159 vs. 0.183, *p* = 0.017). No difference in functional connectivity within Network 1 was observed in the encoding phase. The specificity of group differences was investigated in two additional analyses, where, first, there was no solid group difference in the mean functional connectivity during each memory phase in Network 2 (*p* = 0.081, 0.088, and 0.449 for encoding, maintenance, and retrieval, respectively) and Network 3 (*p* = 0.073, 0.914, and 0.934 for encoding, maintenance and retrieval, respectively). Second, the mean connectivity between the networks (Network 1 and Network 2, Network 2 and Network 3, Network 1 and Network 3) in each memory phase was not different between AD and controls either (all *p* > 0.250, Figure 3).

### AD Had More Nodes With Reduced Connectivity in Network 1 During Retrieval

**Figure 4:**
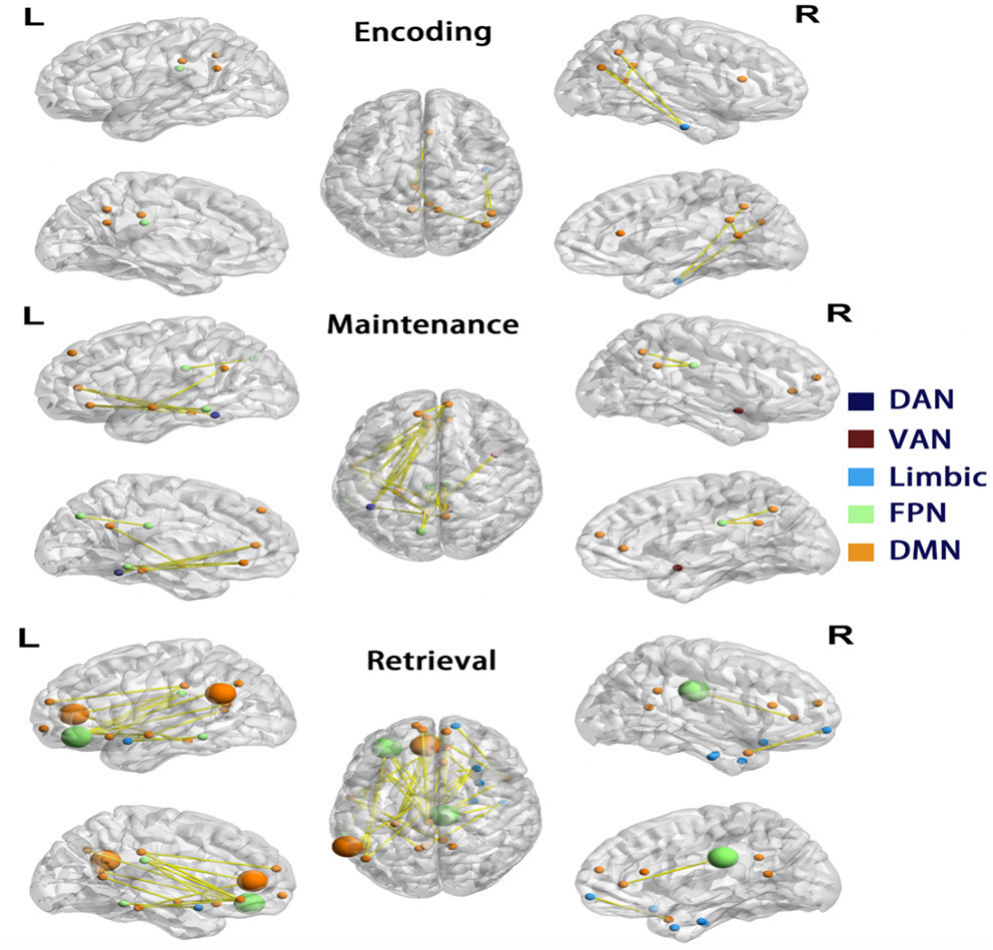
Differences in functional connectivity of Network 1. Each row showed nodes with significantly reduced connectivity in AD compared to controls in each phase, visualized by BrainNet Viewer (Xia et al., 2013). The nodes are colored by the a priori network assignments in the Schaefer atlas. Larger sized nodes indicate significant decreased functional entropy in the AD, as well as decreased functional connectivity. DMN, default mode network; FPN, frontoparietal network; DAN, dorsal attention network; VAN, ventral attention network.

As decreased mean connectivity was found within Network 1 of the AD group during retrieval, node-wise functional connectivity was further compared between AD and controls during encoding, maintenance, and retrieval in Network 1. In AD, during the retrieval phase, 29 in 56 nodes were found to have significantly reduced links (32 edges), and they were located in the medial prefrontal cortex, posterior cingulate cortex, middle temporal and inferior parietal cortex of the left hemisphere, and the inferior temporal and medial prefrontal cortex of right hemisphere. The majority of nodes with reduced connectivity were assigned to DMN (yellow nodes in Figure 4), followed by the limbic network and FPN (blue and green nodes in Figure 4). In the maintenance phase, fewer (16 in 56) nodes were found with reduced links (21 edges) in AD (chi-square *p* = 0.01, compared to the retrieval phase). The reduced connectivity had a similar pattern to that in the retrieval phase, predominantly in the DMN of the left hemisphere. During encoding, only 10 nodes with 9 reduced links in AD (Chi-Square *p* < 0.001, compared with the retrieval phase; *p* = 0.179 compared with the maintenance phase, Figure 4) was observed.

To elucidate the nature of the reduced connectivity in AD, entropy as a measure of BOLD time course predictability (Yao et al., 2013) was investigated in each phase. The AD showed reduced entropy in five nodes (temporal pole, olfactory cortex, medial prefrontal cortex, and inferior prefrontal cortex in the left and cingulate cortex in the right hemisphere) during the retrieval phase, which were all in Network 1 (of these, four nodes had reduced functional connectivity in AD, shown as larger nodes in Figure 4). The results indicated that decreased functional connectivity appeared to be partially characterized by more regular and predictable BOLD time courses in AD during the retrieval phase.

### Relationship Between Cortical Aβ, Network 1 Connectivity, and Cognition

**Figure 5:**
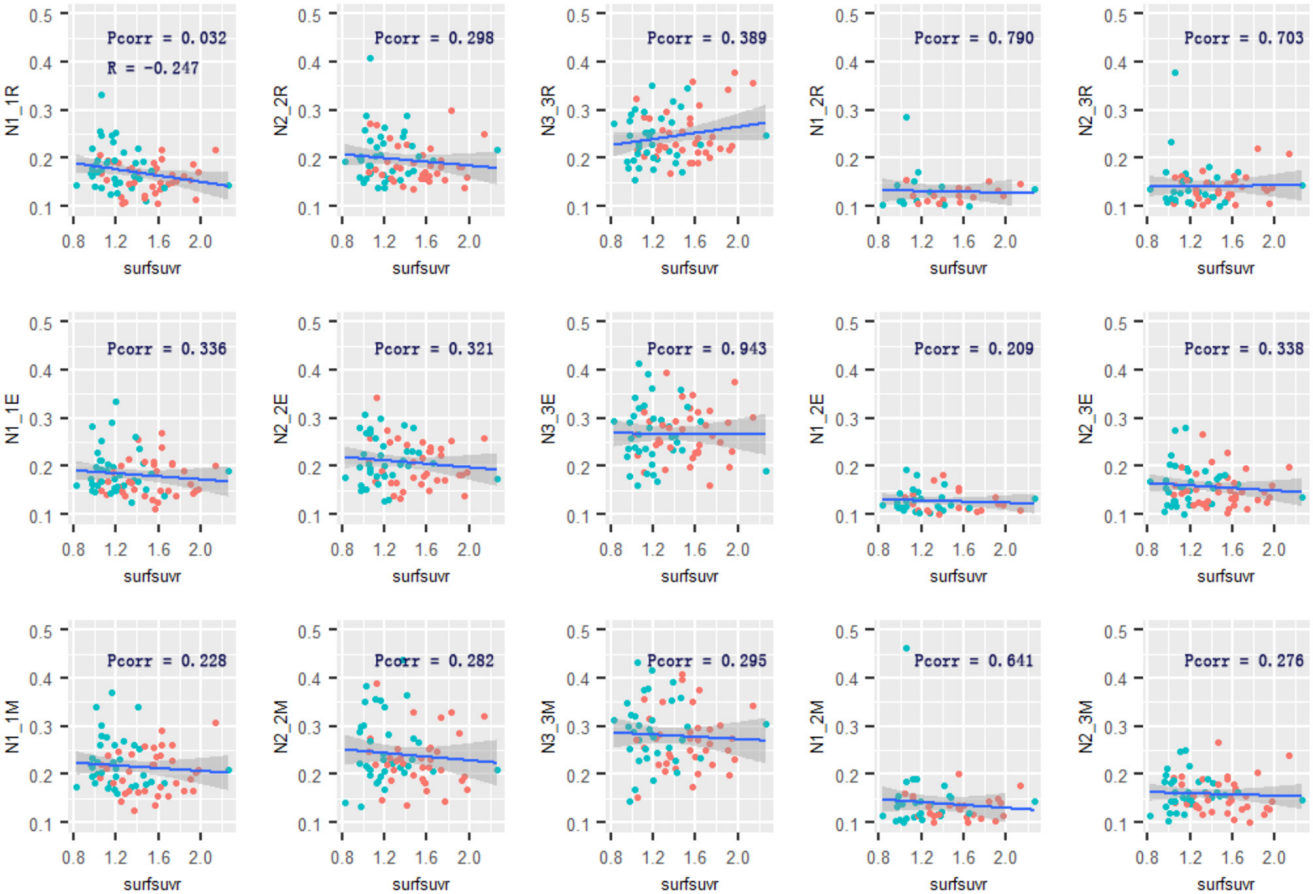
Relationship of cortical Aβ and Network 1–3 connectivity. The cortical Aβ deposition was associated with Network 1-Network 1 functional connectivity in the retrieval phase. No other associations were observed. R and Pcorr represented correlation coefficient and significance across two groups. Pcorr in the correlation analysis was corrected by 10,000 permutations. The gray zone around blue lines represent the 95% confidence interval for predictions from the linear model. surfsuvr = surface standardized uptake value ratios; N1_1, Network 1-Network 1; N2_2, Network 2-Network 2; N3_3, Network 3-Network 3; N1_3, Network 1-Network 3; N2_3, Network 2-Network 3; R, Retrieval; E, Encoding; M, Maintenance.

Across the two groups, there was a negative relationship between global cortical Aβ and the mean connectivity within Network 1 [*R* = −0.247, 95% confidential interval: (−0.453, −0.016), *p* = 0.032] during the retrieval phase. The relationship between Aβ and the mean connectivity during encoding or maintenance was not significant (*R* = −0.113, *p* = 0.336; *R* = −0.142, *p* = 0.228). Only the cortical Aβ had a robust adverse impact on Network 1 connectivity. There was no significant relationship between cortical Aβ and the mean connectivity within Network 2 or Network 3 in any of the memory phases (Figure 5). However, in the AD group, significant relationships between global cortical Aβ and the mean connectivity were not observed within Network 1 (*p* = 0.887) in the retrieval phase, neither in the encoding nor maintenance phases (*p* = 0.746/0.342). The relationships between Aβ and the mean connectivity in the CN group was also not significant (*p* > 0.16).

For completeness, an additional analysis was conducted to test the relationship between Aβ and accuracy-related neural activation during the retrieval phase. Amyloid deposition in activated regions and the global cortex both had a negative effect on task accuracy-related neural activation (*R* = −0.365, *p* = 0.001; *R* = −0.267, *p* = 0.023). In the GLM adjusting age, sex, and education, the cortical Aβ in activated regions had associations with ACER memory (β = −0.43, SE = 0.108, *p* < 0.001), and mean connectivity in Network 1 during retrieval (β = −0.24, SE = 0.116, *p* = 0.043). Noting that the mean connectivity in Network 1 during retrieval associated with ACER memory (R = −0.304, *p* = 0.008), the mediation effect from Aβ on memory was observed by connectivity in Network 1 [mediation effect = −0.08, 95% confidential interval: (−0.15, −0.02), *p* = 0.037]. It suggested the transmission of the effect of Aβ variability on memory through functional connectivity in Network 1.

**Figure 6:**
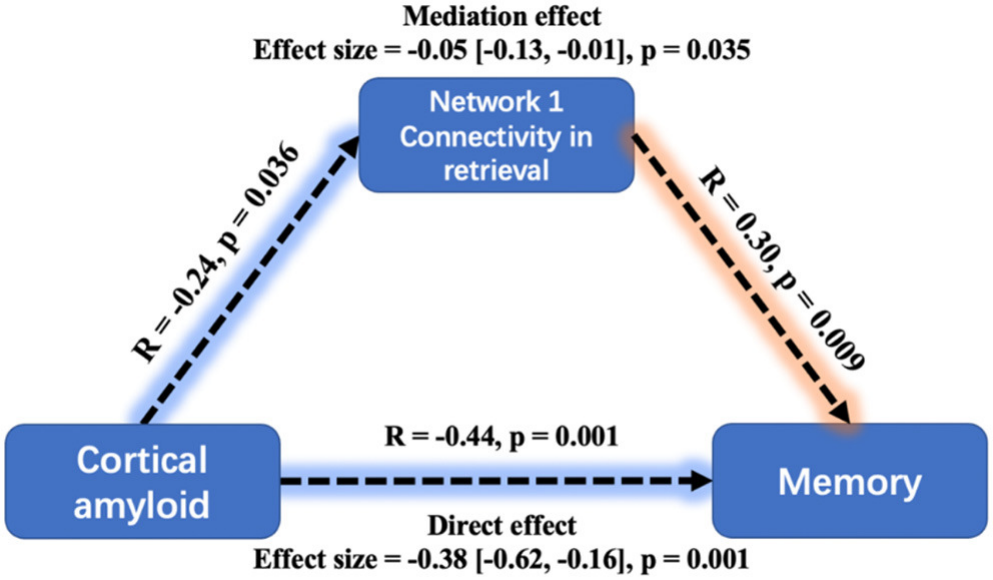
Mediation analysis between cortical amyloid, task-related Network 1connectivity, and memory performance. A greater cortical amyloid deposition has a significant but indirect association with worse memory performance, mediated *via* the mean connectivity during retrieval within Network 1. Mediation effects were computed by 10,000 bootstrapped samples and the 95% confidence intervals were reported.

The global Aβ also had a significantly negative relationship with MoCA (β = −0.34, SE = 0.112, *p* = 0.003) and ACER (β = −0.3, SE = 0.114, *p* = 0.011). Similarly, the mediation analysis indicated that the effect of Aβ on memory performance was mediated through mean connectivity in Network 1 during retrieval: average causal mediation effect = −0.05, 95% confidential interval: (−0.13, −0.01), *p* = 0.035 (Figure 6). The mediation effect was also significant for the effect of Aβ on ACER. However, the mean connectivity within Network 2 or Network 3 had no significant mediation effect on memory and ACER.

## Discussion

It was found that AD participants were mainly characterized by decreased functional connectivities in the Network 1 community (dominantly composed of DMN, the limbic network, and FPN) during the maintenance and retrieval phase in the memory task. In detail, AD had more nodes with reduced connectivity in Network 1 during the retrieval phase than any other phases. The nodes were in the medial prefrontal cortex, posterior cingulate cortex, middle temporal cortex, inferior parietal cortex of the left hemisphere, and inferior temporal and medial prefrontal cortex of the right hemisphere. The global cortical Aβ was associated with decreased mean connectivity in Network 1 only during retrieval. The mediation analysis indicated that cortical Aβ might affect memory performance by modulating retrieval-related functional connectivity within Network 1.

As memory maintenance is believed to require more neocortex regions (Gazzaley et al., 2004, Woodward et al., 2006, Takahama and Saiki, 2014), regions were clustered with assignments to *a priori* networks based on functional connectivity during the maintenance phase. Network 1 was composed of four *a priori* networks, with its regions being spatially well organized in the bilateral temporal cortex, medial prefrontal cortex, posterior cingulate cortex, and occipitotemporal regions (Figure 2). The pattern of Network 1 was like the joint anterior-temporal and posterior-medial system (Libby et al., 2012), which works interactively for encoding and retrieval memory (Collins and Dickerson, 2019, Cooper and Ritchey, 2019). The reduced functional connectivity within Network 1 is consistent with evidence found in resting-state fMRI, both in AD and cognitively unimpaired Aβ+ individuals (Harrison et al., 2019, Berron et al., 2020). Importantly, AD reduced mean functional connectivity, specifically in the maintenance and retrieval phases of memory. As in a previous work, retrieval deficiencies could be the cause of poor performance (Li et al., 2016). An intervention study provided additional evidence for the importance of the retrieval phase in recognition (Smith et al., 2013). Taken together, these suggested that Network 1 successfully clustered regions that were working together for memory maintenance and retrieval, and it was impaired in AD.

Significant positive correlations between the Network 1 connectivity and ACER memory performance were observed in the retrieval phase. The DMN was dominant in Network 1 as it was clustered, and these results are consistent with studies that highlight the importance of DMN activation (Smith et al., 2018) and cortical network reorganization for better cognitive performance (Shine et al., 2016; Shine and Poldrack, 2018), especially when large shifts of attention are required during cognitive operations.

The task accuracy-related activation during retrieval was located in the left superior frontal cortex, within VAN/Network 2. In the working memory model, retrieval, which is generally defined as attention allocation efficiency (Germano and Kinsella, 2005, Wang et al., 2010), requires a phonological loop and central executive. In this study, task activation also correlated with memory performance, suggesting the failure to recruit VAN may also hamper the cognitively demanding selection.

As we found that the hallmark of the AD had decreased mean connectivity in Network 1 during memory retrieval, we further performed node-wise functional connectivity comparisons. As expected from the above results, more nodes had reduced connectivity during the retrieval phase than other phases in AD. Some nodes were clustered in the left posterior cingulate cortex, originally assigned to the left DMN. The DMN being involved in the cognitive process has been studied. During the memory process, the functional connectivity between DMN sub-networks increases in the retrieval of episodic memories (Sestieri et al., 2011). In AD, reduced resting-state functional connectivity was observed in posterior DMN nodes in the precuneus/posterior cingulate cortex (Badhwar et al., 2017). Other than that, another hub in the DMN, the medial prefrontal cortex, was observed to have reduced connectivity. Similarly, previous research on schema memories showed the activation of the medial prefrontal cortex (mPFC) associated with successful memory for schema items (Liu et al., 2017). Importantly, the results showed a strongly left-lateralized connectivity change in the retrieval phase in AD. This fits with earlier studies showing similar left-lateralized effects in early AD using resting-state fMRI (Berron et al., 2020), volumetric gray matter measurements (Shi et al., 2009), FDG-PET (Weise et al., 2018), and amyloid PET (Raji et al., 2008).

To elucidate the nature of decreased functional connectivity in AD, the node-wise entropy in Network 1 was calculated. Nodes with reduced entropy (i.e., reduced BOLD signal predictability) only showed clear spatial correspondence with nodes of reduced connectivity relative to CN during the retrieval phase. This indicated that some regions in Network 1 with decreased functional connectivity were more predictable and less random with reduced complexity. The results revealed retrieval-related connectivity-entropy coupling and showed a difference in AD from normal aging (Yao et al., 2013).

For the adverse effect of amyloid, the negative correlation between cortical amyloid and ACER memory performance was observed. The finding was consistent with previous amyloid studies, which found that higher amyloid deposition correlated with lower immediate memory and delayed recall scores in mild cognitive impairment (MCI) participants and amyloid-positive healthy controls (Doraiswamy et al., 2012; Sperling et al., 2013).

It was once argued that amyloid pathology and neurodegeneration have adverse and, in part, synergistic effects on prospective cognition (Bilgel et al., 2018). In addition to the linear relationship found between Aβ and cognition (Knopman et al., 2018), Aβ load was revealed to have a nonlinear role in moderating the BOLD activation effect on behavioral task performance (Foster et al., 2018, Kennedy et al., 2018). Taken together, these findings suggested the involvement, but lack of direct effect, of Aβ on the cognitive deficiency. Several of these results are in accordance with this hypothesis. First, the correlation between cortical amyloid and memory performance/task activation was not regionally specific. Second, the mediation analyses indicated a significant effect of Aβ on memory performance, and yet mediated through a direct effect on Network 1 connectivity during the retrieval phase. These results suggested that global cortical amyloid might exert an influence on memory performance through an effect on functional connectivity during memory retrieval within Network 1, which mainly included DMN, limbic network, and FPN. Participants with higher cortical amyloid depositions exhibited the pronounced Network 1 connectivity decreases, which were linked to worse memory performance.

The study has some strengths that extend the current literature, including the measurement of cortical amyloid, connectivity, and entropy in the same participants and the examination of memory phase-related functional connectivity. One key limitation is that it was not possible to make strong inferences regarding the direction of causality from purely observational studies. Related to this, there may be unmeasured biomarkers (e.g., tau) and neuronal variables that exert a more direct causal influence on memory performance. These concerns can only be addressed in fully randomized interventional experimental designs. Second, as regionally specific effects from amyloid were not found, findings were the results of the global effects of AD pathology in the cortex. More effort was needed to make sure that the neural activities in certain regions were more vulnerable to local pathological change. Finally, while there were significant correlations between Aβ and connectivity across two groups, their linear relationships were insignificant in AD or the control group, respectively. It could be due to the limited sample size in each group and the few participants in the AD continuum such as MCI. Further work should include participants in different AD stages and explore the effect of Aβ pathology evolution.

## Conclusion

Reduced mean functional connectivity was found in patients with AD. They had more brain regions exhibiting decreased connectivity compared to controls during memory retrieval in the default mode, limbic, and frontoparietal networks. Importantly, it was found that mean cortical Aβ deposition is directly related to the network-specific functional connectivity during the memory retrieval phase. The cortical Aβ may also impair memory performance through its relationship with connectivity. The findings can help in the mapping of impaired functional connectivity during memory phases and explaining Aβ-related memory deficiencies in patients with AD.

## Data Availability Statement

The raw data supporting the conclusions of this article will be made available by the request to the corresponding authors, without undue reservation.

## Ethics Statement

The studies involving human participants were reviewed and approved by The ethical committee of Ruijin Hospital, Shanghai Jiao Tong University School of Medicine. The patients/participants provided their written informed consent to participate in this study.

## Author Contributions

ByL analyzed and interpreted all imaging data and drafted the manuscript. MZ performed PET-MR and was the equal contributor in writing the manuscript. XL, HM, and XH performed PET-MR scanning. WH synthesized the AV45 tracer. GY, LZ, and GH helped with clinical data collection. SC supervised the research. BL and JL supervised, reviewed, and modified the manuscript. All authors read and approved the final manuscript.

## Conflict of Interest

The authors declare that the research was conducted in the absence of any commercial or financial relationships that could be construed as a potential conflict of interest.

## Publisher's Note

All claims expressed in this article are solely those of the authors and do not necessarily represent those of their affiliated organizations, or those of the publisher, the editors and the reviewers. Any product that may be evaluated in this article, or claim that may be made by its manufacturer, is not guaranteed or endorsed by the publisher.
