# Supplementary material for: Amyloid-Beta Influences Memory via Functional Connectivity During Memory Retrieval in Alzheimer's Disease
Source: Front Aging Neurosci. 2021 Sep 1;13:721171. doi: 10.3389/fnagi.2021.721171 (PMC8444623; doi:10.3389/fnagi.2021.721171)
Supplement: Supplementary file 1 [file Data_Sheet_1.docx]

Supplementary Material

# Empirical networks from community detection algorithm

This Louvain algorithm partitions the nodes of the covariance matrix into a community structure that maximizes the modularity of the network, where modularity (Q) is a measure of network segregation and describes the degree to which a network may be subdivided into non-overlapping communities, with the maximal number of within-community connections, and a minimum number of between-community connections (Rubinov and Sporns 2010, Shine, Bissett et al. 2016, Finc, Bonna et al. 2017). For positive weighted networks it is defined as:

$$Q=\frac{1}{v}\sum_{ij} \left( \omega_{ij}-\frac{S_{i}S_{j}}{v} \right)\delta_{M_{i}}\delta_{M_{j}}$$

where $v$ is the total strength of the network ($v=\sum_{ij} \omega_{ij}$), $\omega$_ij_ is the strength of the edge connecting node *i* to *j*, $S_{i}$ is the strength of node *i* ($S_{i}=\sum_{j} \omega_{ij}$), and $\delta$ is set to 1 when nodes *i* and *j* are in the same module, and 0 otherwise (Rubinov and Sporns 2010, Schultz and Cole 2016, Shine, Bissett et al. 2016, Finc, Bonna et al. 2017).

Given the stochastic nature of the Louvain algorithm, we used a consensus clustering approach to ensure the robustness of the final community structure (Lancichinetti and Fortunato 2012, Cohen and D'Esposito 2016, Hearne, Cocchi et al. 2017). Similar to the study (Nour, Dahoun et al. 2019), for each control participant, we iteratively applied the algorithm 1000 times with different initial random seeds. This generated 1000 separate partitions, which were then combined to a single agreement matrix, *D*, where entry *d_ij_* represents the proportion of partitions in which nodes *i* and *j* were assigned to the same community. Following a thresholding step (in which all entries < 50% agreement were set to zero), the agreement matrix was then subjected to another 1000 iterations of the Louvain algorithm. This procedure was repeated until each of the resulting 1000 partitions was equal, resulting in a binary agreement community matrix (i.e. a consensus partition) for each participant. Finally, a mean group agreement matrix was created by averaging the consensus partition agreement matrices of each control participant. This group matrix was then subject to the consensus clustering procedure until a final group community partition was produced. We used the default resolution parameter γ=1.

**Functional connectivity analysis algorithms**

The NBS algorithm is a nonparametric statistical method to deal with the multiple comparisons problem on a graph. The method is used to control the family-wise error rate (FWER), in the weak sense, when performing mass univariate hypothesis testing on all graph edges (e.g. functional connectivity here).

The first step is to independently test the group difference (AD vs CN) at every connection in the network with t-test. Each connection is therefore endowed with a t-value quantifying the evidence in favor of the null hypothesis.

The second step is to choose the t-statistic threshold as the primary threshold. The connections with the t statistic value exceeding this threshold are admitted to a set of supra-threshold connections. Connections comprising this set represent potential candidates for which the null hypothesis can be rejected, although at this stage statistical significance cannot be established. In our study, given AD and control groups, we calculated the network component *C* (defined as a connected set of links), for which *t_ij_* > *t*_0_, where *t_ij_* is the *t*-statistic for the link between (BOLD signal correlation) between node *i* and *j*. The *t*_0_ controls the component size and significance level of constituent links (set to match *P <*0.001 given group sizes). The calculation of all the links’ *t*-values was done in a general linear model framework modeling connectivity as a function of groups and regressing out age and sex.

The third step is to identify topological clusters among the set of suprathreshold connections. In this context, a connected graph component is a set of supra-threshold connections for which a path can be found between any two nodes. All connected components can be efficiently identified with a breadth or depth search.

The final step is to compute a family-wise error rate (FWER)-corrected p-value for each component using permutation testing. The AD and CN groups were permuted for 50,000 times. Each permutation involves randomly permuting the group labels according to a permutation vector containing a random permutation of the integers from 1 to the total number of data points. The same permutation vector is used for every connection to preserve any interdependencies between connections.

For each permutation, the first three steps of the NBS are repeated on the permuted data. In particular, the hypothesis of interest is tested at every connection using the same statistical test, a set of supra-threshold connections is defined using the same threshold and any connected graph components are then identified. The size of the largest component is recorded for each permutation, thereby yielding an empirical null distribution for the size of the largest component size. The component size (number of links) was compared to a permutation-generated null distribution of sizes, thus controlling for the family-wise error rate in the weak sense at α = 0.05.

Applying a two-sided suprathreshold (|*t_ij_*| *> t*_0_), the differences in node-node functional connectivity between two groups would be visualized as binary outcomes in each memory phase.

# Supplementary Figures


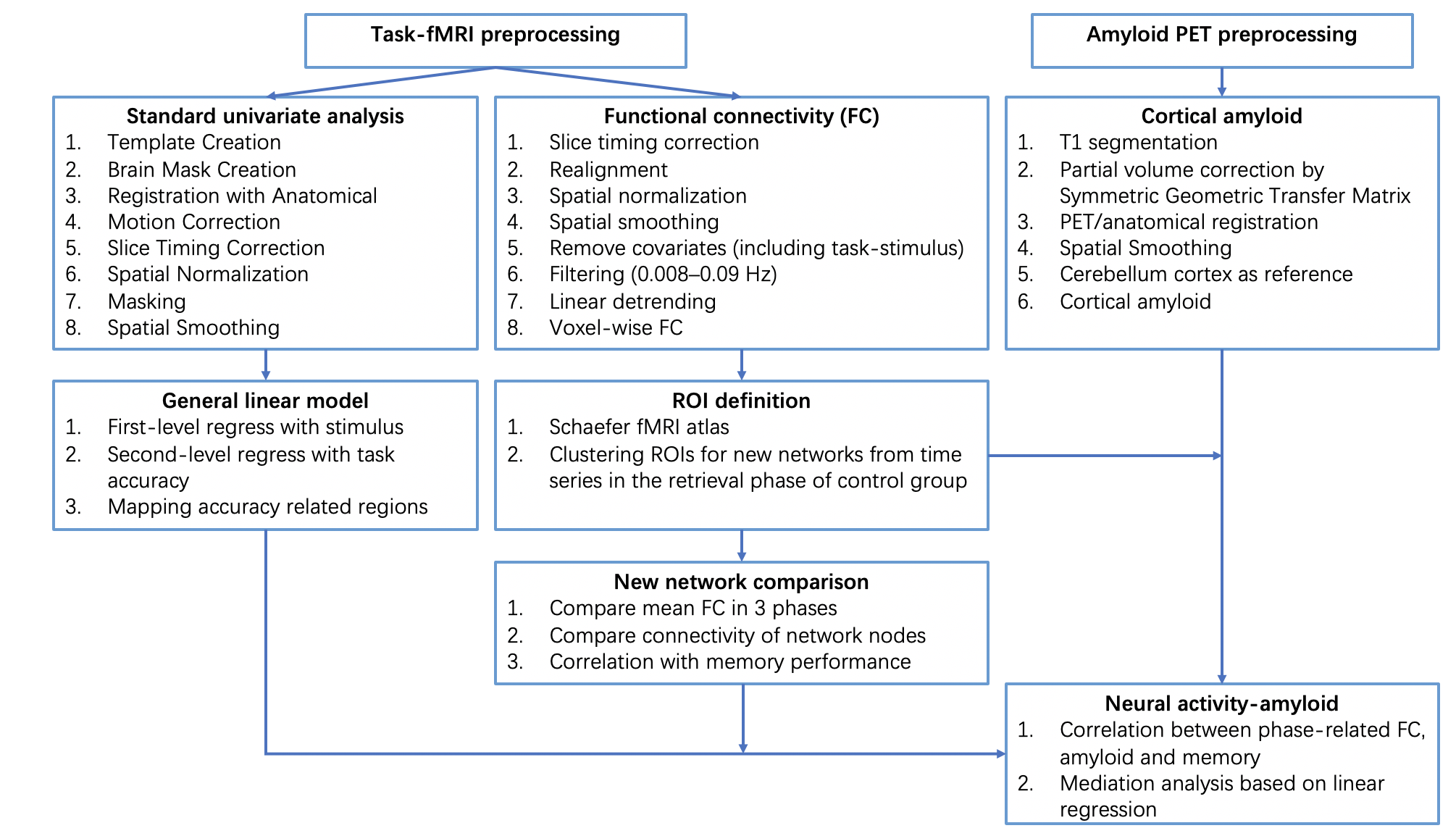


Supplementary Figure.S1 Summary of the analysis approach

Reference

Cohen, J. R. and M. D'Esposito (2016). "The segregation and integration of distinct brain networks and their relationship to cognition." Journal of Neuroscience 36(48): 12083-12094.

Finc, K., K. Bonna, M. Lewandowska, T. Wolak, J. Nikadon, J. Dreszer, et al. (2017). "Transition of the functional brain network related to increasing cognitive demands." Hum Brain Mapp 38(7): 3659-3674.

Hearne, L. J., L. Cocchi, A. Zalesky and J. B. Mattingley (2017). "Reconfiguration of brain network architectures between resting-state and complexity-dependent cognitive reasoning." Journal of Neuroscience 37(35): 8399-8411.

Lancichinetti, A. and S. Fortunato (2012). "Consensus clustering in complex networks." Sci Rep 2: 336.

Nour, M. M., T. Dahoun, R. A. McCutcheon, R. A. Adams, M. B. Wall and O. D. Howes (2019). "Task-induced functional brain connectivity mediates the relationship between striatal D2/3 receptors and working memory." eLife 8.

Rubinov, M. and O. Sporns (2010). "Complex network measures of brain connectivity: uses and interpretations." Neuroimage 52(3): 1059-1069.

Schultz, D. H. and M. W. Cole (2016). "Integrated Brain Network Architecture Supports Cognitive Task Performance." Neuron 92(2): 278-279.

Shine, J. M., P. G. Bissett, P. T. Bell, O. Koyejo, J. H. Balsters, K. J. Gorgolewski, et al. (2016). "The Dynamics of Functional Brain Networks: Integrated Network States during Cognitive Task Performance." Neuron 92(2): 544-554.
